# Constructing Genetic Networks using Biomedical Literature and Rare Event Classification

**DOI:** 10.1038/s41598-017-16081-2

**Published:** 2017-11-17

**Authors:** Amira Al-Aamri, Kamal Taha, Yousof Al-Hammadi, Maher Maalouf, Dirar Homouz

**Affiliations:** 1 Department of Electrical and Computer Engineering, Khalifa University of Science and Technology, P.O. Box 127788 Abu Dhabi, UAE; 2 Department of Industrial and Systems Engineering, Khalifa University of Science and Technology, P.O. Box 127788 Abu Dhabi, UAE; 3 Department of Physics, Khalifa University of Science and Technology, P.O. Box 127788 Abu Dhabi, UAE

## Abstract

Text mining has become an important tool in bioinformatics research with the massive growth in the biomedical literature over the past decade. Mining the biomedical literature has resulted in an incredible number of computational algorithms that assist many bioinformatics researchers. In this paper, we present a text mining system called Gene Interaction Rare Event Miner (GIREM) that constructs gene-gene-interaction networks for human genome using information extracted from biomedical literature. GIREM identifies functionally related genes based on their co-occurrences in the abstracts of biomedical literature. For a given gene g, GIREM first extracts the set of genes found within the abstracts of biomedical literature associated with g. GIREM aims at enhancing biological text mining approaches by identifying the semantic relationship between each co-occurrence of a pair of genes in abstracts using the syntactic structures of sentences and linguistics theories. It uses a supervised learning algorithm, weighted logistic regression to label pairs of genes to related or un-related classes, and to reflect the population proportion using smaller samples. We evaluated GIREM by comparing it experimentally with other well-known approaches and a protein-protein interactions database. Results showed marked improvement.

## Introduction

In the post-genomics era, researchers have realized the importance of studying protein interactions to understand the biochemical pathways. The most reliable practice to determine these interactions is through experimental methods that discover the protein’ s functions and use them in the understanding of biological processes. Most advance experimental techniques can’ t keep up with the rapid growth in the size of biological knowledge to be studied. Thus, the need has arisen for faster and reliable prediction systems. Predicting the protein/gene interactions for the whole genome (i.e. human genome, yeast genome, etc.) results in constructing genetic networks. Several computational systems have been developed to predict the protein functions by using the protein sequence or protein structure^1–3^. Tiwari *et al*.^4^ provides an extensive review on various computational techniques for protein function predictions using sequence and structure data. The massive growth of new biological data leads to similar increase in biomedical literature and biomedical data. To date, PubMed -which is a database of indexed biomedical literature citations- holds more than 27 million citations^5^. The growth of the literature also accompanied the arising interest in the research of literature-mining to extract biomedical relations.

Text mining has become an important process for analyzing and extracting patterns from the biomedical literature^6,7]^. Text mining is somehow considered a branch of Data mining despite being different in few aspects^8^. They both in general extract knowledge; where the knowledge is about data in the case of data mining and text features in the case of text mining. Text mining presents a higher level of difficulty compared to data mining in the sense that the text is unstructured and usually harder to study. Nevertheless, different text mining applications have been implemented to extract the relations between biological entities. Extracting patterns -that represent genes or proteins- from the literature needs to be recognized and presented in a useful way to be understood and employed in the prediction of protein/gene functions. Studying the connections in genetic networks help biologist to reveal different functions of the genome and the type of interactions between biological entities^9,10^. Understanding the genes behaviors is crucial in the further discovery of gene-disease and gene-drug associations^11^. Thus, determining genetic networks by using biomedical published text provides a great alternative and effective aid for biologists to keep up with the rapid research in the genetic area^12^.

The main techniques for the biological entities extracted from the literature are summarized into co-occurrence techniques^13,14^, rule-based systems and machine-learning-based systems^15–17^. Co-occurrence approaches determine the genes that are related by their co-occurrence frequency found in the literature. These approaches also usually adopt some weighting schemes to further distinguish the relation type and rank the relations extracted. For example, Sharma *et al*.^18^ proposed a binary relation extraction method using the sentence level. They use rules mapped from sentence dependency tree to represent each pair of terms (biological entities) found in a sentence as a vector of features. This method then classifies these vectors using *Support Vector Machine* (SVM).

Text miners with biological interest also target the identification of several biological entities and not necessary protein or gene names alone^19–22^. Gene Ontology (GO) terms are utilized widely in the implementation of text mining methods^23–25^. For example, Text-KNN approach proposed in^26^ determines the functions of genes by extracting their names and GO terms from the biomedical literature. This approach represents each gene found in an abstract by the GO terms associated with it. Text-KNN assigns each GO term with an occurrence probability and consider the highest probabilities to describe a certain gene. Using a k-nearest neighbor classifier, Text-KNN annotates genes whose functions are unknown with functions of annotated genes that share the same GO terms.

In this paper, we propose an approach called **G**ene **I**nteraction **R**are **E**vent **M**iner (GIREM) that aims at constructing a relation network for the human genome “*H*. *Sapiens*”. It does so predicting the connected genes based on the co-occurrence of genes and the GO terms within the literature. This approach contributes to the state-of-the-art biomedical text mining approaches by effectively utilizing the information extracted at both the abstract level and the sentence level. We show the importance of having the knowledge of both levels by having the following assumption; A pair of genes (g1, g2) that appear together in N sentences and R abstracts is considered to be less related than another pair (g1, g3) that appear together in N sentence but D abstracts (where D > R). We also present a novel approach in using a supervised machine learning algorithm (weighted logistic regression) to recognize related pairs where each is represented by a vector of features.

The co-occurrence of a pair of genes in a sentence may not be an indicative of the association between the two genes. Therefore, GIREM automatically extracts from biomedical abstracts each co-occurrence of a pair that represents *semantic relationship* between the pair of genes. Towards this, we adapt some linguistic discovery techniques (i.e., semantic rules) that identify the semantic relationship between each co-occurrence of a pair of genes using the syntax and the structural linguistics principles^21,27^. GIREM constructs genetic network after extracting the set of pairs that appear in the same sentence and share a semantic relationships.

## Problem Statement and Outline of the Approach

One of the key goals of biological Natural Language Processing (NLP) is the automatic information extraction from biomedical publications. Despite their huge potential, the most current approaches that construct genetic networks and deploying text-based terms suffer from the following limitations:

1. They select gene terms by considering solely the number of their occurrences in abstracts. Others consider only the occurrences in sentences. As a result, some of these terms lack the certainty and could be misclassified to wrong functional classes.
2. Most of these approaches overlook the semantic relationships between the terms (*biological entities*) within a sentence.
3. They don’ t study the long-distance relation between terms occurring in two different sentences.

GIREM uses NLP for the automatic information extraction from biomedical publications and overcomes most of the limitations of current approaches outlined above. Specifically, GIREM aims at constructing a gene-gene-interaction network for the human genome by identifying genes mentioned in the PubMed/MEDLINE abstracts. It consists of the following four main components:

- Information extraction component.
- The gene representation component.
- The classification component.
- The prediction component.

**Figure 1:**
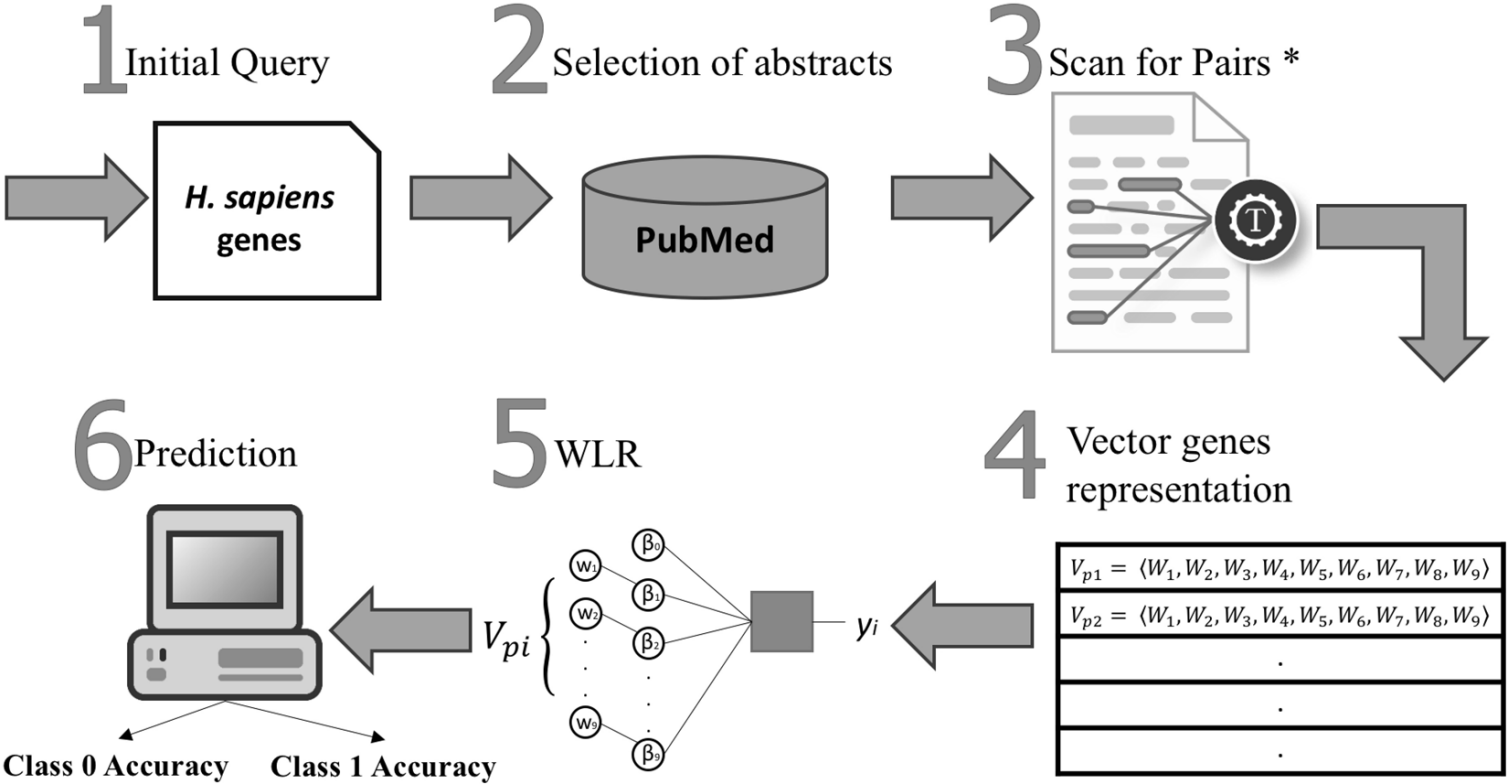
The sequential steps taken by the system. *GIREM extract the co-occurrence of pairs of genes at three level; the abstract, the sentence and the semantic level.

The detailed steps of our approach are illustrated in Fig. 1. GIREM applies novel computational methods and semantic techniques to break the limitations of some of the current approaches outlined above, as follows. It determines the *semantic relationship* between each pair of biological terms in a sentence using semantic rules. It does so by applying an *information extraction model* that mines the semantic relation. The model examines the linguistic units to extract and state the relationship between two terms.

The following is an overview of our approach, in terms of the sequential processing steps taken by GIREM to construct genetic networks:

1. **Selecting genes and GO Terms for GIREM:** We select a set of genes annotated with reliable functions from a high-quality biological database such as UniProtKB/Swiss-Prot^28^. The selected set of genes and the GO terms assigned to them will be used as a dataset for training GIREM. Each of the selected genes should satisfy the following two conditions: (1) should be annotated with at least one GO category, and (2) the entry of the genes in the biological database should have at least one reference to a PubMed text.
2. **Downloading the PubMed texts associated with the genes selected in step 1:** We download the primary names for the *H*. *Sapiens* genes and their GO term associations. We then used the set of genes to retrieve the PubMed abstracts that mention at least one gene. The National Center for Biotechnology Information (NCBI)^5^ includes various databases and e-utilities that we have used to download all the PubMed abstracts and save them in a local database. The two main e-utilities that we were used are “e-search” to search the PubMed IDs associated with a certain gene, and “e-fetch” to retrieve and download the PubMed abstract using the previously downloaded PubMed ID^29^.
3. **Information extraction component:** GIREM automatically extracts each co-occurrence of a gene-molecule pair that represents semantic relationship between the pair. A molecule in the framework of GIREM is a gene or a GO term. Towards this, we present semantic rules that identify the semantic relationship between each co-occurrence of a gene-molecule pair using the syntax (study of sentence sturctures) and the structural linguistics principles. GIREM locates and identifies the associations that describe semantic relationships between a pair of genes using dependency parsing and information extraction techniques. These techniques rely on the structures of sentences and linguistic principles. We present efficient information retrieval mechanisms that examine the associations that exist between pairs of genes in the large amount of biomedical literature associated with the pair. *This process is described in more details in section* “*Information Extraction Component*”.
4. **Feature**-**representation component:** In the framework of GIREM, a 9-dimensional vector of variables is used to represent each pair of genes (*P*_*i*_) found in the literature. Each of these variables is a feature that shows the co-occurrence percentage either in the abstract level, the sentence level or the semantic level. The first three variables in the vector describe the co-occurrence percentages (*i*.*e*. *abstract*, *sentence*, *semantic*) for the two genes in the pair *P*_*i*_. The second three variables -middle three- in the vector describe the co-occurrence percentages for the first gene in *P*_*i*_ with the GO terms of the other gene (second gene) as retrieved from UniProtKB. The last three variables describe the co-occurrence percentages for the second gene in *P*_*i*_ with the GO terms of the first gene. The nine variables construct the vector of weights and total resulted vectors are referred to as *X*. *This process is described in more details in section* “*Feature*-*representation Component*”.
5. **The classification component:** The resulted table of vectors X construced in step 4 is fed to a weighted logistic regression (WLR) classifier to identify the probablity of pairs of genes being related. GIREM uses Bootstrapping to identify gene functions. In general, bootstrapping is a re-sampling method used to obtain estimates of summary statistics. It generates large number of samples and offers a simple and true assessment of the error measure^30^. This method works by randomly selecting a sample from the set of testing data going on a number of rounds. The final accuracy is obtained by taking the average of the total accuracies scored at each round. *This process is described in more details in section* “*The Classification and The Prediction Component*”.
6. **The prediction component:** GIREM assigns the class for each pair of genes based on the probability of forming an interaction. The parameters of this probability are obtained by maximizing the log-likehood function in the WLR classifier. *This process is described in more details in section* “*The Classification and The Prediction Component*”.

## Information Extraction Component

We select a set of annotated *H*. *Sapiens* genes as well as their annotation GO terms from a reliable biological database such as UniProtKB/Swiss-Prot. We then retrieve the PubMed abstracts associated with these genes that are referenced in the entry of the biological database. GIREM extracts from these abstracts the genes and GO terms that co-occur with the selected genes. It automatically extracts from the retrieved abstracts each co-occurrence of a pair of genes and gene-GOterm that represents semantic relationship between the pair. The information of these pairs will be used as text features to represent selected genes. Our objective is to represent these genes using text features that are highly predictive of their potential functions. GIREM concludes that the two entities in the pair (gene-gene) or (gene-GOterm) are related as follows:

- It reports the summation of co-occurrences of two genes or a gene and a GO term in the abstracts. It also separately reports the summation of co-occurrences in the sentences.
- It tracks the individual occurrences of a gene or a Gene Ontology (GO) term in abstracts and in the sentences.

Based on the results of the extraction we calculate the ratio of the co-occurrences over the total number of individual occurrences. This ratio is computed for the abstracts level, sentences level and -as later on explained- the semantic level.

First, GIREM employs Named Entity Recognition (NER) technique. NER is the automated identification of relevant terminology from texts (e.g., gene names). It relies on two components (i.e. tokenizer and stemmer) to parse each word in the sentence and detect the names of molecules. The co-occurrence of a gene’s name and a molecule’ s name in a sentence or an abstract may not be a definite indication of the association between the gene and the molecule. Therefore, GIREM automatically extracts from biomedical abstracts each co-occurrence of a gene-molecule pair that represents semantic relationship between the pair. Towards this, we implement some of the association discovery techniques (i.e., semantic rules) that discover the semantic relationship between each co-occurrence of a gene-molecule pair using the syntax and the structural linguistics principles.

GIREM utilizes some semantic rules for extracting *gene relationships* information from biomedical abstracts. It applies a linguistic model for extracting the functional relationship from different structural forms of terms in the sentences of biological abstracts. Specifically, GIREM extracts phrases that represent functional relationships between genes and genes-GO terms. Genes and GO terms that are related to a given gene g are highly predictive of the functions of g. GIREM represents each gene by the molecules that have high co-occurrences *with the gene* in biomedical abstracts. We determine whether the mentioning of two genes in a sentence represent semantic relationship based on the following syntax and structural linguistics theories:

1. *Sentences Containing Coordinating Conjunctions*: Our main semantic rules utilize these observations^21,26,31^: (1) two phrases or clauses seperated by a contrasting conjunction (e.g. “but”, “whereas”, etc.) are considered opposing and unrelated, and (2) two clauses connected by a similarity conjuntion like (“and”, “or”, etc.) are usually considered related. Toward these observations, we propose the following rules:

- A molecule and a gene pair are considered semantically unrelated if they occur in the same sentence and they are separated by a contrasting conjunction. Therefore the co-occurrence of the two terms in that sentence is not considered.
- The co-occurrence of a molecule and a gene pair in a sentence where they are connected by a similarity conjunction is considered semantically related.

2. *Sentences Containing other linking words*: Some words or phrases express addition, result, reason, etc. (e.g. “As a result”, “As well as”, “as”) to indicate a similarity connection between two clauses^32^. Other phrases can be observed to show directly the relation between two biological entities such as (“binds to”, “interacts with”, “associates”). We retrieved these words and phrases to add them to our proposed rules:

- A molecule and a gene pair are considered semantically related if they are connected within a sentence by one of the above linking words.
-  Moreover, we overlook the linking word if a contrasting conjunction appears together with a linking word. In this case we consider the genes unrelated. In the example “*geneA binds with geneB but not with geneC*”, geneA is semantically related to geneB, and geneA is not semantically related to geneC.

## Feature-representation Component

Our primary goal is to represent each pair of genes using terms (i.e., genes and GO terms) that are highly predictive of their potential association. Each pair of genes is represented by a vector of weights. Each weight represents the co-occurrences of a characteristic term (i.e., a gene or a GO term) in the set of abstracts of PubMed associated with the pair. That is, each weight quantifies the likelihood of the association between a pair of genes and a characteristic term based on the occurrences of the characteristic term in the set of abstracts of PubMed associated with one of the genes in the pair.

In the framework of GIREM, we employ a 9-dimensional vector of weights to represent each pair of genes *g*_*x*_ and *g*_*y*_ found in the biomedical abstracts. We will refer to these weights as *variables or predictors* throughout the paper. Each of the nine variables represents either the direct or the indirect co-occurrences of the two genes. We look at the occurrences at three levels, i.e. the abstract level, the sentence level and the semantic level –sentences that show semantic relation-. The first three variables in the vector describe the co-occurrence frequency for the two genes in the pair *P*_*i*_ at the levels (abstract, sentence, and semantic). The second three variables -middle three- in the vector describe the co-occurrence frequency also at three levels for the first gene in *P*_*i*_ with the GO terms of the other gene (second gene) as retrieved from UniProtKB. The last three variables describe the co-occurrence frequency for the second gene in *P*_*i*_ with the GO terms of the first gene.

An informative description of the first three variables with their matching equations is shown as follows: The first three predictors represent the co-occurrence ratio for the two genes *g*_*x*_ and *g*_*y*_ in the abstracts level, the sentences level and the semantic level respectively. We adopt some of the Information Extraction techniques proposed in PPFBM^21^ for representing the semantic similarity between the two genes in the pair *P*_*i*_. This is interpreted by the understanding of the coherence of the sentence that mentions both genes. We look for words and phrases linking both genes in a positive or a negative connection. We proposed certain rules (*recall section* “*Information Extraction Component*”) to follow and to infer the semantic relation between two terms. *W*_1_, *W*_2_, and *W*_3_ share similar equations where the difference is in the summation limit within the equations.

For each pair of genes mentioned in the biomedical abstracts, we calculate the number of times that both genes occur together. The co-occurrence status (0 or 1) of both genes *g*_*x*_ and *g*_*y*_ in *α*(*i*) is represented by *co*(*g*_*x*_, *g*_*y*_)_*i*_, where *α* indicates the level of occurrence and |*α*| indicates the total (*α* = *A* when we are studying the abstract level and so |*A*| is the number of abstracts that were analyzed, *α* = *S* to indicate the sentences level, and *α* = *SE* for indicating the sentences that show a semantic relation). The mentioning status (0 or 1) of *g*_*x*_ in *α*(*i*) is referred to as “solo occurrence” *so*(*g*_*x*_)_*i*_ and likewise for *g*_*y*_. We used the following equation to compute the first three predictors. *N* = 1, 2, *or* 3

1 $${W}_{N}=\frac{{\sum }_{i=1}^{|\alpha |}co{({g}_{x},{g}_{y})}_{i}}{{\sum }_{i=1}^{|\alpha |}so{({g}_{x})}_{i}+{\sum }_{i=1}^{|\alpha |}so{({g}_{y})}_{i}}$$

Equation 2 shows the example of calculating *W*_1_ where this predictor targets the abstract level and the direct relation between two human genes (TP53 and BRCA1).

*N* = 1, *α* = *A*, |*A*| *is the number of abstracts that either genes appear in*, *g*_*x*_ = *TP*53, *g*_*y*_ = *BRCA*1

2 $${W}_{1}=\frac{{\sum }_{i=1}^{|A|}co\,{(TP\mathrm{53,}BRCA1)}_{i}}{{\sum }_{i=1}^{|A|}so\,{(TP53)}_{i}+{\sum }_{i=1}^{|A|}so\,{(BRCA1)}_{i}}$$

The previous three variables/predictors describe the co-occurrence ratio for the *direct mention* of two genes in the abstract level, the sentence level and the semantic level. The middle three and the last three predictors (*W*_4_–*W*_9_) are constructed similarly however the difference is that these variables describe the *indirect mention* i.e. the co-occurrence ratio between the first gene and the GO terms of the second gene, and vice versa. Since Gene Ontology is a key bioinformatics source for the annotations of proteins/genes, we extract the GO terms from the biomedical literature and include them in our weighting scheme. The algorithm extract the GO terms from the literature whether it’ s a mention of the Gene Ontology term or the GO ID.

The following two equations show the weighing scheme for predictors (*W*_4–6_) and (*W*_7–9_). *W*_*N*_ in equation 3 denotes the co-occurrence ratio in the three levels between the first gene (*g*_*x*_) in the pair *P*_*i*_ and the GO terms (as a group) for the second gene (*g*_*y*_). In this case the co-occurrence status is denoted by *co*(*g*_*x*_, *GOs*(*g*_*y*_))_*i*_. *so*(*g*_*x*_)_*i*_ refers to the solo occurrence of *g*_*x*_ as denoted previously. Identifying a GO term (*GO*_*k*_) results in updating the solo occurrence of the GO terms for all the genes that are annotated by *GO*_*k*_ [*i*.*e*. *GOs*(*g*_*x*_), *GOs*(*g*_*y*_), *GOs*(*g*_*z*_), *etc*]. *N* = 4 for abstract, *N* = 5 for sentence and *N* = 6 for semantic, and *α* denotes the level as in equation 1.

3 $${W}_{N}=\frac{{\sum }_{i=1}^{|\alpha |}co\,{({g}_{x},GOs({g}_{y}))}_{i}}{{\sum }_{i=1}^{|\alpha |}so\,{({g}_{x})}_{i}+{\sum }_{i=1}^{|\alpha |}so\,{(GOs({g}_{y}))}_{i}}$$

*W*_7–9_ are exactly as *W*_4–6_, however the denotation here is between the second gene (*g*_*y*_) in the pair *P*_*i*_ and the GO terms (as a group) for the first gene (*g*_*x*_). *N* = 7, 8, *or* 9

4 $${W}_{N}=\frac{{\sum }_{i=1}^{|\alpha |}co\,{(GOs({g}_{x}),{g}_{y})}_{i}}{{\sum }_{i=1}^{|\alpha |}so\,{(GOs({g}_{x}))}_{i}+{\sum }_{i=1}^{|\alpha |}so\,{({g}_{y})}_{i}}$$

This information extraction algorithm generated a table of pairs (vectors) each represented by nine predictors. We will refer to the table of vectors as *X*. An Example vector resulting from the information extraction algorithm and indicating the variables for *P*_*i*_: ***i*** *denotes the* ***i*** *th pair*

$${X}_{i}=\langle {W}_{1},{W}_{2},{W}_{3},{W}_{4},{W}_{5},{W}_{6},{W}_{7},{W}_{8},{W}_{9}\rangle $$

## The Classification and The Prediction Component

The resulted table of vectors, matrix(*X*) is fed to the weighted logistic regression (WLR) classifier^33^. The reason behind choosing this classifier is to predict the rare events of gene-gene connections with reasonable sample size^34^. Incorporating weight in logistic regression (LR) would reflect the population proportion of the events and non-events in the sample. In both models (LR and WLR), the vector of predictors is represented in a logit transformation function defined by $ln\,(\tfrac{{p}_{i}}{1-{p}_{i}})={X}_{i}\beta$ where *X*_*i*_ is a row vector in *X*, *p*_*i*_ in this case is the probability of the pair of genes being related, *β* is a vector of parameters that differentiate the event and the non-events (the positive class and the negative class). The difference between the two models is present in estimating the log-likelihood where it is expressed as follows in the simple LR. [*y*_*i*_ is 1 if the *i*th training example pair was related and 0 otherwise, and *n* is the total number of training examples].

5 $$lnL\,(\beta )=\sum _{i=1}^{n}\,ln\,(\frac{{e}^{{y}_{i}{x}_{i}\beta }}{1+{e}^{{x}_{i}\beta }})$$

The log-likelihood is maximized to get the unknown vector *β* that leads to the best separation between the events and non-events. The log-likelihood for WLR (shown in equation 6) is adjusted using the weight *w*_*i*_ that represents the proportion of events to non-events. The weight in logistic regression introduces rare event classification, and since we are dealing with imbalanced data problem, we choose to follow the method by Maalouf ^33^ to represent the sparsity of data. The weight is also represented by ${w}_{i}=\tfrac{Q}{H}$ where *Q* represents the proportion in the population and *H* represents the proportions in the sample. The ratio $\tfrac{Q}{H} < 1$ when the proportion of events in the sample is more than that in the population. In this case, the events are given less weight. On the other hand, non-events are given more weight if their proportion in the sample is less than that in the population.

6 $$lnL\,(\beta )=\sum _{i=1}^{n}\,{w}_{i}ln\,(\frac{{e}^{{y}_{i}{x}_{i}\beta }}{1+{e}^{{x}_{i}\beta }})$$

In addition, we used a regularization parameter (*λ*) to avoid singularities and overfitting^34^. *λ* affects the bias and variance behaviors. In general, a large value of *λ* results in high bias and low variance which means that the model couldn’ t determine the correct data behavior and missed the important features resulting in a weak prediction model. A small value of *λ* however, leads to low bias and high variance meaning that the determined trend of data is complex and more fluctuated that the actual data which causes random noise in the prediction. In this work we use bias correction described in (Maalouf & Sidiqi^34^) to account for any bias resulting from regularization, small samples, and rare events. The *λ* parameter is tuned using a bootstrapping method that is explained further at the end of this section.

7 $$lnL\,(\beta )=\sum _{i=1}^{n}\,{w}_{i}ln\,(\frac{{e}^{{y}_{i}{x}_{i}\beta }}{1+{e}^{{x}_{i}\beta }})-\frac{\lambda }{2}{\Vert \beta \Vert }^{2}$$

We train GIREM using a database that provides the information of experimentally verified related genes as a benchmark. Examples of well-known sources for experimental data are STRING^35^ and, KEGG Pathways^36^. We assigned the value “1” to indicate that the pair of genes represented by *X*_*i*_ are verified to be experimentally related according to a verified source. However, assigning the value “0” to indicate non-relation was not as definitive as assigning value “1” as it is usually hard to determine the non-relation between two genes. Towards this, we followed an assumption that will indicate the non-relation and we explain this in details in *section* “*Training Data and Optimal Parameters*”. GIREM uses Bootstrapping to tune the regularization parameter and produce the best fit parameters. Bootstrapping is a simple random sampling method that generates accuracy measures at different rounds and help in checking the stability of systems.

The process of training is the process of finding the *β* vector that better separates the positive and negative class. This procedure depends on the values of (*λ*), that each produces a different vector values of *β*. Typically, regularization improves the overall performance of the model and stabilize the noise level in our data, however, only using the optimal guess value of *λ* will do. We compared the predicted *y* (0 or 1) using the resulted *β* values with the previously assigned *y* using the “experimentally related genes” database. The assigned class is predicted as follows:

8 $${y}_{i}=\{\begin{array}{ll}0, & P({y}_{i}|{X}_{i}\beta )\le 0.5\\ 1, & P({y}_{i}|{X}_{i}\beta ) > 0.5\end{array}$$

As previously stated *X*_*i*_ is a vector of predictors and *β* is a vector of parameters that assign each pair of genes or vector to either class 0 (un-connected) or class 1 (connected). And so, by predicting the connected genes we are able to construct the human genetic network.

## Training Data and Optimal Parameters

In this section we give a description of the results achieved from the previous sequential steps taken by GIREM. We downloaded the primary names for the *H*. *Sapiens* genes and their GO term associations from UniProtKB^28^ which is a catalog of information on proteins and genes. We downloaded a total set of 20,183 genes and used them to download the PubMed abstracts that mention at least one gene. We retrieved a total of 7,894,920 abstracts and saved them into a local SQL database. GIREM starts by extracting the Homo sapiens genes that are mentioned in each abstract from the database of abstracts. Following these steps we fed the abstracts to the information extraction algorithm which resulted in a total of 423,142 pairs (vectors) each represented by nine predictors.

We trained GIREM using the STRING^35^ database as a benchmark. STRING holds information derived from many sources i.e. experimental data and computational prediction approaches. It scores the protein-protein-interaction (PPI) out of 1000, and reference these interactions using gene names. We retrieved all the *H*. *Sapiens* PPIs using the STRING API, and isolated the experimental data using the experimental score parameter “escore”. All the retrieved PPIs were saved to a local database and compared to the table of pairs we constructed previously (*recall section* “*Feature*-*representation Component*”). We assigned *y*_*i*_ to 1 for all the pairs (row vectors in *X* represented by *X*_*i*_) that are verified to be experimentally related according to STRING database.

It is not deterministic, however, to assign *y*_*i*_ to “0” for the pair of genes that are not experimentally related according to STRING DB. We made the assumption of assigning *y*_*i*_ to 0 for the pair of genes (vector) that don’ t have a common gene interaction within the PPI network. This technique resulted in assigning 20,469 pairs to the positive class (related) and 317,084 to the negative class (non-related). The rest of the 85,589 pairs were used later in the prediction phase. We have added the values of indicating the class to the set of predictors each vector has, resulting in a representation of 9 variables with the relation indication. Table 1 shows an example representation of the data fed to the classification.

**Table 1:** Example Data fed to the Classification System.

Pairs	Vector (predictors)	Relation
g1-g2	<0.5, 0.29, 0.29, 0.5, 0.3, 0, 0.2, 0.14, 0>	1
g2-g3	<0.3, 0.22, 0.21, 0.24, 0.1, 0, 0, 0, 0>	0
g1-g4	<0.5, 0.5, 0.5, 0.4, 0.3, 0, 0.2, 0.1, 0>	1
g2-g5	<0.3, 0.22, 0.21, 0.24, 0.1, 0, 0, 0, 0>	0
g3-g4	<0.3, 0.15, 0, 0.13, 0.1, 0, 0, 0, 0>	0
g4-g5	<0.4, 0.25, 0.21, 0, 0, 0, 0, 0, 0>	0

We evaluated GIREM by testing the accuracies (for both class 0 and class 1) and by setting the number of “*bootstrapping*” rounds to 1000. We created a random training set of 30,000 vectors with their relation *y*_*i*_ (equal data sets of 15,000 representing each class). The optimal regularization parameter(*λ*) was found by testing values varying from [0–10000] with the same training set, and by evaluating the accuracy with each value. The weighted logistic regression classifier generated the optimal *β* that separates the two classes (class 0 and class 1) at *λ* that scored high accuracies for both classes. The optimal *β* parameters and optimal regularization parameter *λ* are indicated in Table 2.

**Table 2:** Optimal *β* parameters and optimal regularization parameter *λ*.

*λ* value	4328
*β* parameters	$$\begin{array}{rcl}\beta & = & \langle -2.0\times {10}^{-4},2.5\times {10}^{-2},1.5\times {10}^{-2},1.4\times {10}^{-2}\\ & & -1.7\times {10}^{-4},-2.1\times {10}^{-5},-1.2\times {10}^{-5}\\ & & 3.0\times {10}^{-6},1.8\times {10}^{-5},1.3\times {10}^{-5}\rangle \end{array}$$(9)

The *β* vector values indicates the weight given to each predictor. WLR decides on the *β* parameters values by observing the relevant features (predictors) in the training data. Some predictors show more relevance to the relation between genes and some are considered not relevant upon training the data. A positive sign indicates that the parameter has a strong relevance in determining if a pair of genes are connected, and on the other hand, a minus sign indicates weak relevance to the relation between genes in the pair.

**Figure 2:**
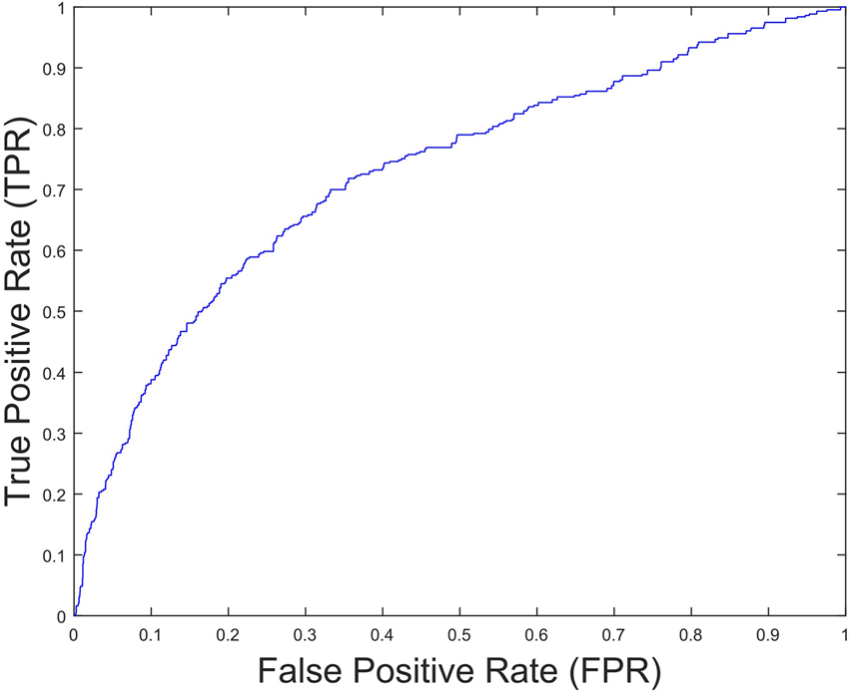
ROC curve for Training data. In this case TPR is increased at low FPR.

We performed a search over the regularization parameter space to optimize the accuracy measure for both classes. We tested the system’ s measures of True positives and True negatives as they are the most relevant measures to ascertain that the WLR model represents the best quality of the system. We achieved a high accuracy for both classes as seen in Table 3. Obtaining high accuracies for both classes individually rather than their average is crucial for the assurance of the best separation in training data and it will also have a good effect on the prediction of testing data. We show the Receiver Operating Characteristic (ROC) curve in Fig. 2 to assess the quality of our system.

**Table 3:** Accuracy measure from training data of 30,000 pairs of genes.

	Accuracy	AUC
class 0 (unrelated)	**68**	**74**
class 1 (related)	**68**

## Experimental Results

We implemented GIREM in Java, and we run it on Intel(R) Core i7 processor, with a CPU of 3.4 GHz and 16 GB RAM, under Windows10. We used Ling Pipe APIs for the information extraction algorithm and implemented the classification model in MATLAB. GIREM determines the interactions among the genes in the entire human genome, based on the analysis of their co-occurrence with each other and with GO terms in the PubMed abstracts. GIREM used a total of 7,894,920 abstracts to identify the genes interactions. All the retrieved abstracts are associated with at least one human gene.

### Evaluating the Accuracy of Identifying Related Genes

We evaluated the accuracy of the system in terms of its Recall and Precision for identifying related genes, using two different datasets as benchmarks: STRING^35^ and KEGG^36^.

#### STRING testing data set

**Figure 3:**
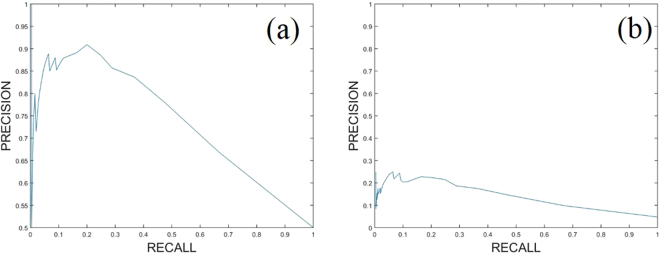
Precision-Recall Curve for (**a**) balanced data and (**b**) imbalanced data.

GIREM identified 423,142 pairs/vectors (*recall section* “*Feature*-*representation Component*”). To predict their relation we trained GIREM with 30,000 pairs and by using STRING as a benchmark. Here we are running a prediction test for a set of 10000 pairs that were not used in the training process and by using the optimal *β* weights we obtained from WLR classifier. We evaluated the prediction accuracy of GIREM by identifying the true positives and false positives according to STRING. Results are shown in Table 4. This test used a balanced data set for testing (equal number of pairs from class 0 and class 1). In Fig. 3 we show how GIREM balances both recall and precision for balanced and imbalanced data sets.

**Table 4:** GIREM Accuracy Measures using different datasets.

	*Recall*	*Precision*	*F*-*value*	*TNR*
STRING	**67**	**67**.**5**	**67**.**3**	**68**
KEGG	**25**	**36**.**5**	**30**	**56**

#### KEGG testing data set

We also calculated the Recall and Precision by testing the pairs (vectors) against the KEGG pathways. We used the measures we obtained using WLR to predict the positive class and the negative class. Similar to the STRING test, we run the prediction test for a set of 10000 pairs and we compared these predictions with the KEGG dataset to compute the true positives and false positives. The KEGG dataset consider related genes as follow: Genes that appear in the same pathway are considered related according to the definition of pathways. As for class 0, we made a similar assumption to the one we followed in section “*Training Data and Optimal Parameters*”. Genes that do not appear in the same pathway are considered un-related but both genes in the pair have to be appearing at least once in one of the pathways. Measures on recall, precision, f-value and True Negative Rate (TNR) are shown in Table 4. The measures for both datasets (KEGG and STRING) were computed using true positives (*TP*), false positives (*FP*) and false negatives (*FN*):

$$\begin{array}{llllll}\,Recall & = & TP/(TP+FN) & Precision & = & TP/(TP+FP)\\ Fvalue & = & \frac{2\times Recall\times Precision}{Recall+Precision} & \quad \,\,TNR & = & TN/(TN+FP)\end{array}$$

GIREM was trained using STRING as a benchmark, hence the higher accuracy measures compared to KEGG as seen in the following table. In addition, the amount of interactions retrieved from KEGG pathways were more than 10 times higher than STRING.

### Comparing GIREM with other Approaches

We also evaluated GIREM by comparing its prediction accuracy with those of (**1**) BioGRID^37^ and (**2**) GOSemSim package^23^.

1. *BioGRID*: BioGRID stands for The Biological General Repository for Interaction Datasets. It is a publicly available database that holds -up to date- more than 1,400,000 protein/gene interactions from different organisms and species, and which is mainly derived from the biomedical literature. This database provides an easy access to individual users via a REST service. Interactions data can be retrieved programmatically by using various parameters and can be viewed in different formats.
2. *GOSemSim*: It is an R package that computes the semantic similarity among genes and GO terms. These computations are executed by implementing the five approaches which are Wang method^38^, Resnik^39^, Lin^40^, Rel^41^ and Jiang^42^. We used the GOSemSim package to compare GIREM with the mentioned five methods. Out of the six main functions provided by the package, we used ***mgeneSim*** to compute the semantic similarity among a list of genes. We were able to compare GIREM with each of the five methods as the function ***mgeneSim*** parameters allow the user to specify which method to use in calculating the semantic similarity.

#### Tests and Benchmarks

**Figure 4:**
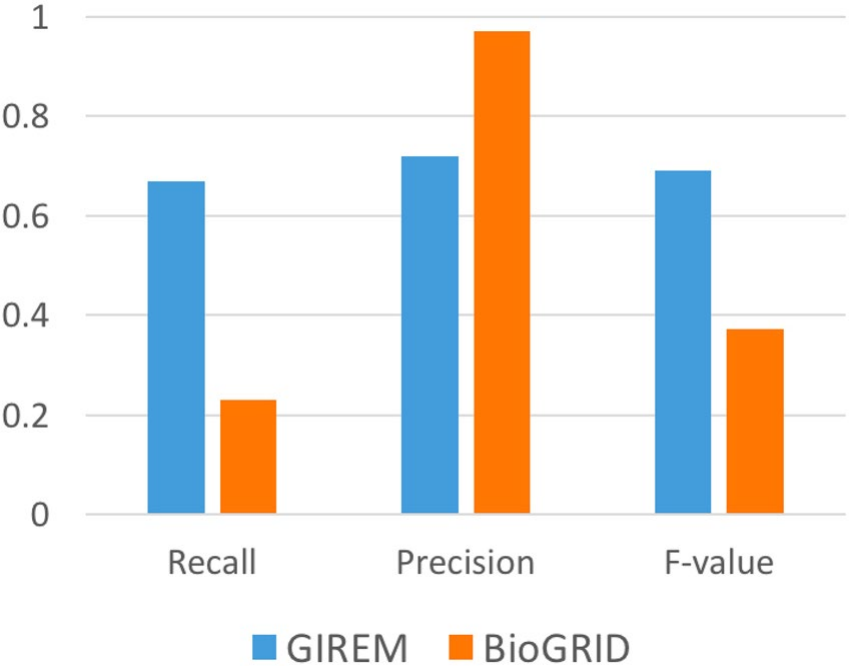
The overall average Recall, Precision, and F-value over several rounds of tests and by using STRING as a benchmark.

*With BioGRID*: In this test, we evaluated the performance of GIREM and BioGRID using as a benchmark the STRING biological database. STRING holds protein interactions from various sources (i.e. experimental data and prediction systems). We only retrieved the interaction data that is based on experimental sources. We then used it to evaluate and compare GIREM resulted dataset with BioGRID interactions. We ran several tests each with a different sample size. Out of all the constructed pairs by GIREM, over 20000 pairs were verified to be related according to STRING. We covered a wide range of these pairs in this test and predicted their relation according to GIREM and BioGRID. Samples sizes for the different tests covered 10–20000 pairs that are related according to STRING and another 10–20000 pairs that are not related to create a balanced sample test. We compute the recall, precision and f-value for each round and report the average results in Fig. 4.

An example test is as follows. We chose randomly *n* pairs that are considered related according to STRING and predicted these pairs using GIREM and BioGRID. We predicted the *n* pairs according to GIREM by using the optimal *β* vector found in section “*Training Data and Optimal Parameters*”. We also tested each pair by retrieving the related pairs according to BioGRID. The Related Pairs according to each system is referred to as *RP*_*system*_. The accuracy measures were calculated as follows:

$$\begin{array}{rcl}\quad \,Recall & = & ((R{P}_{system}\cap n)/n)\\ Precision & = & ((R{P}_{system}\cap n)/R{P}_{system})\quad F\,value=\frac{2\times Recall\times Precision}{Recall+Precision}\end{array}$$

*With GOSemSim*: In this test, we evaluated the performance of GIREM and GOSemSim by also using STRING biological database as a benchmark. For this test we randomly selected 15 genes to create a sample space of (15 × 15) pairs of genes. Each of these pairs were assigned to related or un-related classes based on the benchmark. We predicted the relation of each pair according to the five methods in GOSemSim package and according to GIREM, and compared these relations to the ones from the benchmark. An Example test is as follows. The number of related pairs out of the total 225 pairs according to STRING are *R*_*STRING*_. Similarly, the related pairs according to one of the methods in GOSemSim (e.g. Wang) is *R*_*Wang*_. The performance measure were computed as follows:

$$\begin{array}{rcl}\quad \,\,Recall & = & (({R}_{Wang}\cap {R}_{STRING})/{R}_{STRING})\\ Precision & = & (({R}_{Wang}\cap {R}_{STRING})/{R}_{Wang})\end{array}$$

**Figure 5:**
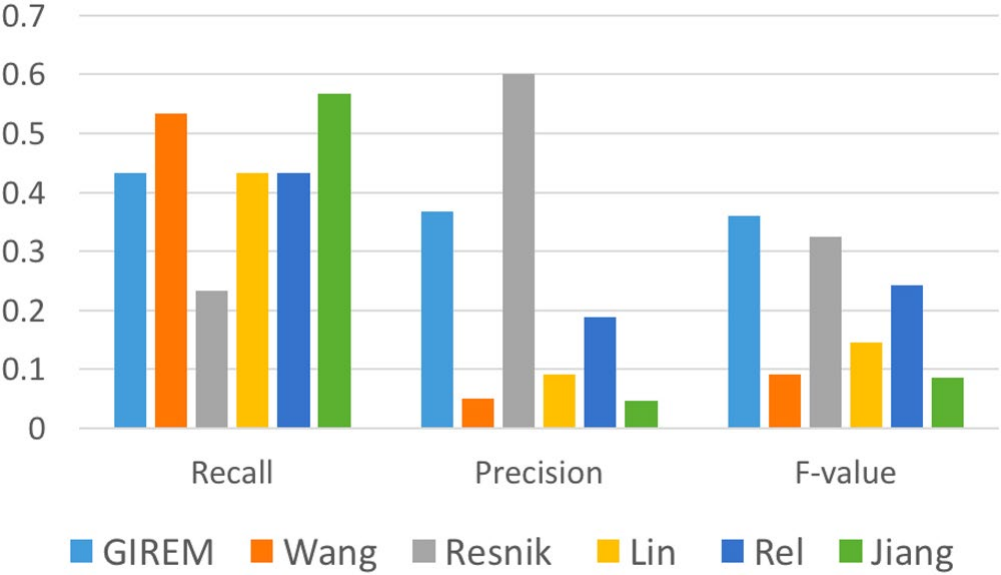
Accuracy measures of GIREM and the five methods in GOSemSim package for determining functionally related genes.

We repeated this test several times each with a random list of 15 genes. We report the average results in Fig. 5.

In general, the majority of the limitations in our data set that we could consider to increase the accuracy for class 0 and 1 as well as the recall and precision were as follows: (1) we only considered the primary names of *H*. *Sapiens* genes, and in many abstracts the authors refer to genes’ names using the synonyms as well. (2) Our linguistic model doesn’ t consider the long distance relation between genes or gene-GOterms as the algorithm looks at each abstract sentence at a time. (3) A very likely source of limitation is the assumption we followed to assign a pair of genes to class 0 if they are not experimentally related according to the STRING GGI network.

## Conclusion

In this paper, we presented a text mining approach to construct the genetic network of the human genome “*H*. *Sapiens*”. We predicted the relation for a pair of genes by extracting and analyzing their co-occurrence in the biomedical literature. We gave an overview of the approach main components; the information extraction algorithm, the gene representation component, the classification and the prediction components. Our approach identified the related genes using supervised machine learning algorithm (Weighted Logistic Regression). WLR classified the pairs of genes to either classes (class 0 and class 1) and by testing them against experimentally verified related genes from STRING database. Our Approach achieves high accuracies for both classes with an AUC = 74%. In addition, we compared our method with other well-known approaches and a protein-protein interactions database.

We plan to extend our work by overcoming the limitations in our data set to increase the overall accuracies of the classes and the precision. Specifically, we intend to use more descriptive linguistic theories to allow for a better extraction of the genes relation. Another direction of work is the further study of gene-disease associations and link them to the genetic network constructed in the current work.
